# Role of Epigenetics in the Pathogenesis, Treatment, Prediction, and Cellular Transformation of Asthma

**DOI:** 10.1155/2021/9412929

**Published:** 2021-09-15

**Authors:** Binaya Wasti, Shao-kun Liu, Xu-Dong Xiang

**Affiliations:** ^1^Pulmonary and Critical Care Medicine, The Second Xiangya Hospital, Central South University, Changsha, Hunan 410011, China; ^2^Diagnosis and Treatment Center of Respiratory Disease, Central South University, Changsha, Hunan 410011, China; ^3^Research Unit of Respiratory Disease, Central South University, Changsha, Hunan 410011, China

## Abstract

Asthma is a mysterious disease with heterogeneity in etiology, pathogenesis, and clinical phenotypes. Although ongoing studies have provided a better understanding of asthma, its natural history, progression, pathogenesis, diversified phenotypes, and even the exact epigenetic linkage between childhood asthma and adult-onset/old age asthma remain elusive in many aspects. Asthma heritability has been established through genetic studies, but genetics is not the only influencing factor in asthma. The increasing incidence and some unsolved queries suggest that there may be other elements related to asthma heredity. Epigenetic mechanisms link genetic and environmental factors with developmental trajectories in asthma. This review provides an overview of asthma epigenetics and its components, including several epigenetic studies on asthma, and discusses the epigenetic linkage between childhood asthma and adult-onset/old age asthma. Studies involving asthma epigenetics present valuable novel approaches to solve issues related to asthma. Asthma epigenetic research guides us towards gene therapy and personalized T cell therapy, directs the discovery of new therapeutic agents, predicts long-term outcomes in severe cases, and is also involved in the cellular transformation of childhood asthma to adult-onset/old age asthma.

## 1. Introduction

Asthma is well known as a noncommunicable, chronic, and heterogeneous inflammatory condition of the lower airway tract characterized by various clinical conditions that vary in severity and frequency [1, 2]. Asthma can be observed in any age group. Some epidemiological studies have shown that asthma begins early in preschool age, although symptoms appear later in adult life [3] and, in some cases, may not appear.

The common asthma phenotypes are type 2 asthma (T2 high) and non-type 2 asthma (T2 low). T2 asthma includes early-onset allergic and late-onset nonallergic eosinophilic asthma. The common Th2 biomarkers used in clinical practice are mainly blood eosinophils, fractional exhaled nitric oxide (FeNO), and IgE levels. Interestingly, the majority of people with T2 asthma respond well to standard therapy with inhaled corticosteroids [4]. However, non-T2 asthma is a neutrophilic and paucigranulocytic heterogenous type, predominant in those with adult-onset and corticosteroid-resistant (less responsive), and inflammation-driven through IL-17, IL-6, and IL-23. Further, it has airway smooth muscle or neural dysfunction and may be associated with comorbidities, such as obesity and gastroesophageal reflux disease [5, 6]. The exact linkage between childhood and adult-onset/old age asthma remains unclear. Does adult-onset/old age asthma represent the persistence or relapse of childhood asthma?

Although we have ongoing research and an improved understanding, prevalence of asthma has been increasing in recent years [7] and affecting more than 339 million people worldwide [8], with 417,918 deaths globally in 2016 [9]. Asthma is a heritable disease [10], and approximately 60% of heritability has been found in several studies [11]. Interestingly, monozygotic twins are four times more likely to develop asthma than dizygotic twins (19% vs. 4.5%) [12]. Despite many studies, the complete natural history, pathogenesis, and heterogeneous phenotypes of asthma remain unclear. These unsolved questions and increasing asthma incidence suggest that there may be other elements related to asthma pathogenesis with heredity, leading to researchers investigating the connection between epigenetic changes and asthma.

Waddington described the term epigenetics more than half a century ago [13]. Later, Nanney described it as an unexplainable inherited phenomenon by conventional genetics [14]. In 2007, it was defined preciously using three criteria: (I) genetic changes without mutation, (II) initiation by a signal (extracellular signal), and (III) inheritance by mitosis or meiosis [15]. Currently, epigenetics is regarded as a significant influencing factor in asthma pathogenesis [16], and many epigenetic studies involving asthma are currently underway. Commonly studied epigenetic phenomena include DNA methylation, histone modification, and small noncoding RNA (miRNAs). We hope to obtain a clear understanding of asthma through epigenetic studies in the near future.

Despite extensive knowledge, there is still much unknown about asthma. There are many new etiologic factors, variations in presentation, and new genetic linkage with asthma. Therefore, we must ask if there are any new factors driving asthma development and progression besides the known factors and etiology. This review is aimed at providing an overview on asthma epigenetics and its components, including asthma epigenetic studies, and discuss potential epigenetic links between childhood asthma and adult-onset/old age asthma.

## 2. Can Asthma Epigenetics Be Considered a Hot Research Topic?

**Figure 1:**
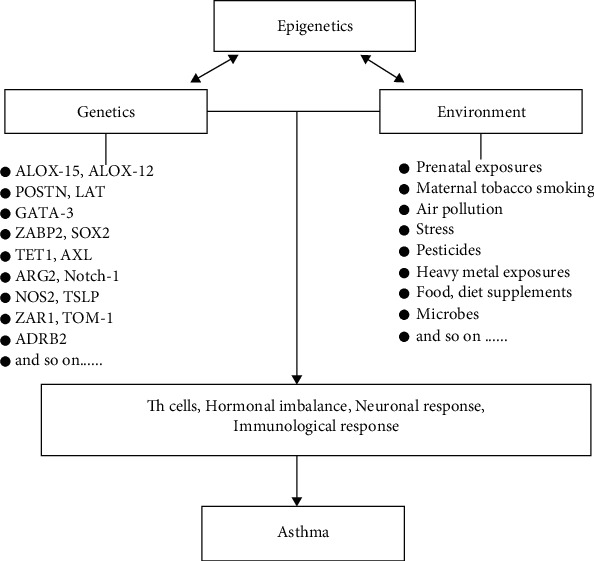
A schematic view showing the association between epigenetic mechanism and genetic and environmental factors in asthma. Genetics and environment are regulated by epigenetic factors. However, it is known that this interaction is more complex, and environmental factors, as well as genetic factors, can also regulate epigenetics. Several other components of the immune, neuronal, and hormonal response are also involved in the outcome. Th cells: T helper cells.

The answer is yes. Asthma epigenetics have received immense interest because genetic and environmental factors cannot wholly and independently explain asthma etiology, heterogeneity, and phenotypes (Figure 1). Researchers' search to find an alternative explanation for still mysterious phenotypes and heterogeneity of asthma made asthma epigenetics a hot topic. Moreover, the results are providing answers to the above queries.

## 3. DNA Methylation

Historically, DNA methylation in mammals was documented just after the discovery of DNA as a genetic component [17]. Methylation is the addition of a methyl group, by DNA methyltransferase, on cytosine at position 5 with the formation of 5-methylcytosine [18] where guanine nucleotides follow the cytosine nucleotide known as CpG [19]. Approximately, 60%-80% of CpG islands (cluster of CpG) are usually methylated in humans, but not in single CpG islands [20]. Interestingly, this equals to only 1% of bases and 5% of cytosine methylation across the gene [21]. CpG islands usually contain more than 200 bases with approximately 50% guanine plus cytosine (G+C) and a ratio of 0.6 or more [22]. Methylation of CpG island results in gene activation or inhibition but usually promotes repression, since the islands are located in the transcription start site (TSS) of genes [23]. Recently, our study found that methyl CpG binding domain protein 2-mediated Th17 differentiation in severe asthma is associated with IRF4 and SOCS3 expression, providing novel insight into epigenetic regulation and target for treatment in severe asthma [24, 25]. A specific term “epimutation” is used in literature to denote the heritable genetic changes caused by DNA modifications [26].

Bisulfite conversion (sequencing) of DNA is the most commonly used technique for DNA methylation. Other assessment techniques include high-performance liquid chromatography, microarrays or bead chips, mass spectrometry, methyl sensitive cut counting (MSCC), and antibodies or protein that bind to methylated DNA [27]. To date, the DNA methylation techniques have only covered 450000 (450K Infinium Human Methylation Beadchip, Illumina Inc., CA, USA) and 850000 (Infinium Methylation EPIC Beadchip, Illumina, USA) CpG through the epigenome-wide association study [28, 29]. We hope that the methylation technique will be developed further to allow for the capture of more CpG positions at once.

### 3.1. DNA Methylation and Asthma

**Figure 2:**
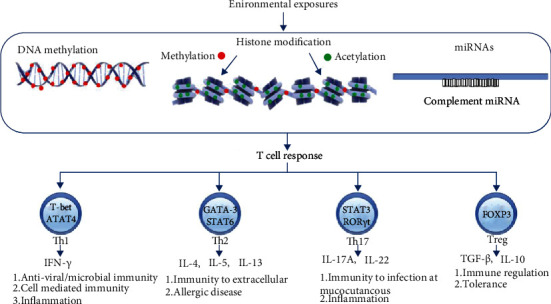
The role of epigenetics in the pathogenesis of asthma. An epigenetic mechanism links genetic and environmental factors with developmental trajectories in asthma. The most commonly studied asthma epigenetic phenomena are DNA methylation, histone modifications, and miRNAs. DNA methylation is the process of adding methyl groups, by DNA methyltransferase, on cytosine at position 5 with the formation of 5-methylcytosine [18] where guanine nucleotide follows the cytosine nucleotide known as CpG [19]. The methylation of CpG islands (clusters of CpG) results in gene activation or inhibition, but usually repression, because the islands are found almost near the genes' transcription start site (TSS) [23]. Histone modification usually occurs at N-terminal with possible modifications on each “basic” residue, but common residue targets for modifications are lysine, serine, arginine, and tyrosine threonine. Acetylation, methylation, phosphorylation, ubiquitination, and sumoylation are well-known histone modification mechanisms. Histone acetylation and histone methylation are the most studied and better known to us [82]. Histone acetyltransferases (HATs) and histone deacetylases (HDACs) work in opposition to each other as acetylation by HATs favors gene expression and deacetylation by HDACs is responsible for gene silencing. The addition of phosphate, i.e., phosphorylation, is mediated by kinases, while phosphatases remove the phosphate. Similarly, histone ubiquitination is mediated by ubiquitin ligases and opposed by ubiquitin-specific peptidases, also known as deubiquitinating enzymes. Histone sumoylation is mediated by the histone sumoylation proteins, also known as the small ubiquitin-like modifier (SUMO) protein. A microRNA (miRNA) is a 22-25 nucleotide single-stranded small noncoding RNA molecule transcribed from the DNA. However, miRNAs are not translated into proteins and play a role in gene expression either by blocking or by altering mRNA translation stability [86]. Alteration in epigenetic status results in differential gene expression related to cytokines and transcription factors, resulting in various and distinct phenotypic presentations in asthma. Some T cell subsets and cytokine production and functions are also shown in the figure. miRNAs: micro-RNAs; Th cells: T helper cells.

DNA methylation is the most studied mechanism in asthma. Alteration in DNA methylation status results in differential gene expression related to cytokines and transcription factors, resulting in various and distinct phenotypic presentations in asthma (Table 1 and Figure 2).

*ALOX15* (genetic loci: 17p13.2) and *POSTN* (genetic loci: 13q13.3) genes were hypomethylated in the nasal epithelium and associated with asthma [30]. *ALOX15* promotes eosinophilic inflammatory diseases, such as asthma [31], and aspirin exacerbated respiratory disease (AERD) [32]. The *POSTN* gene is upregulated in asthmatic airway epithelium by Th2 cytokines and can be used as a biomarker for long-term predictability in the management of severe asthma [33]. The increased risk of persistent wheezing resulted from hypomethylation of *ALOX12* in blood, at genetic loci 17p13.1 [34]. Hypermethylation of CpG sites related to *FOXP3* (12q15) and *IFN-γ* (Xp11.23) results in impaired T cell function along with the repression of Tregs and T effector cell genes in the blood [35]. Asthma is associated with hypomethylation of *IL-4*, *RUNX3*, and *TIGIT* in blood, at genetic loci 5q31.1, 1p36.11, and 3q13.31, respectively [36].

Hypermethylation of *IL-2 Site1* (4q27) has been found in the cord blood of children with severe asthma who were followed up for 8 years [37]. Hypomethylation of *IL5RA* (3p26.2) in the blood is associated with asthma in teens [38], and IL-5 plays a crucial role in eosinophilopoiesis [39]. Hypermethylated *GATA3* (location 10p14), a significant regulator of Th2 differentiation, is associated with reduced asthma risk in cord blood [40], while *ZPBP2* (Zona Pellucida Binding Protein 2) hypomethylation results in asthma in a lymphoblastoid cell line at genetic location 17q12-q21 [41]. *CYP26A1* (10q23.33), a regulator of the allergic immune response, was found to be hypermethylated in blood samples of patients with asthma [42]. Hypomethylation of *TET1* (10q21.3) from the nasal epithelium results in asthma in children exposed to traffic-related air pollution [43]. *TET1* converts 5-methylcytosine to oxymethylcytosine along with 5-formylcytosine, 5-carboxylcytosine, and 5-hydroxymethylcytosine, resulting in DNA demethylation. IL-36, IL-38 [44], and IL-3 are believed to be new members of the IL-1 family and are involved in the pathogenesis of asthma, and hypermethylation of *IL1R2* (2q11.2) in blood shows asthma-related phenotypes [45]. These studies show the various phenotypic presentations of asthma through the methylation of specific asthma-related genes.

Increased risk of wheezing, especially in girls, is associated with hypermethylation of the *AXL* gene (19q13.2) in CpG islands in newborn blood spots [46]. Hypermethylation of arginase 2 (14q24.1) results in decreased FeNO in the buccal cells [47], and airway epithelium hypomethylation of nitric oxide synthase 2 (17q11.2) and IL-6 (7p15.3) is associated with an increased concentration of FeNO [48].

The *ADRB2* gene (5q31-33) sampled from blood is hypermethylated and associated with severe asthma, but nitrogen dioxide (NO_2_), a known air pollutant, has been shown to alter this association [49]. Interestingly, one study has shown decreased dyspnea episodes in asthmatic children with *ADRB2* hypomethylation sampled from blood and saliva [50]. Hypermethylation of protocadherin (*PCDH20*) has been found in the sputum of asthmatic adult smokers at the genetic locus 13q21.2 [51]. Epigenetic changes in the beta-2-adrenergic receptor (*ADRB2*) gene play a vital role in the phenotypic presentation of asthma.

Differently methylated regions are considered the beginning of development stages and with reprogramming progress [58]. Cadherin-related genes are differently methylated in children with atopy and atopic asthma [52]. Cadherin-related genes *CDH26* and *CDHR3* (genetic loci 20q13.33 and 7q22.3, respectively) from the epithelial airway are hypomethylated in children with a history of atopy and can easily differentiate atopy with atopic asthma [52]. The noticeable hypomethylation has been found in genes *C7orf50* and *ZAR1* (genetic loci 7p22.3 and 4p11, respectively), which are sampled from cord blood and are associated with IgE levels [53]. *STAT5A* genes are downregulated, differently methylated, and associated with different asthma levels [54]. *STAT5A* genes in the region 17q21.2 are hypermethylated and associated with enhanced Th1 response and decreased eosinophil recruitment in the airway epithelium [54]. These methylation studies show the usefulness in identifying IgE association, eosinophil recruitment, Th1 response, and even differentiating between children with atopy and atopic asthma through the epigenetic changes observed in respective genes at respective loci.

Research has shown that asthmatics showed distinctive methylation differences associated with the *WNT* signaling gene in neutrophilic asthma and purine and calcium metabolism gene in eosinophilic asthma. Hypermethylation of *WNT2* genes in the region 7q31.2 from blood samples is associated with neutrophilic asthma [55]. Similarly, a distinctive and specific methylation profile was observed with FeNO, eosinophil count, and inhaled corticosteroid in asthmatic adults [56]. Hypermethylation of *ORMDL3* from endobronchial airway epithelial cells at the genetic locus 17q12-21 results in asthma in adults [56]. Hypermethylation of IL-13 from airway epithelial cells at location 5q31 is seen in asthma [57]. From these studies, we can conclude that a distinctive and specific methylation pattern is associated with asthma phenotypes and endotypes.

### 3.2. Environmental Exposures, DNA Methylation, and Asthma Risk

The environment is considered a significant influencer of DNA methylation and asthma. Maternal tobacco smoking, air pollution, stress, heavy metal exposures, pesticides, microbes, and possibly some foods are the epigenetic influencer. Therefore, the epigenetic mechanism, through the influence of environmental factors, affects the gene transcription and results in a more complex phenotypic pattern and presentation of asthma and allergy [59].

Prenatal and early childhood maternal tobacco smoking is considered a risk factor for asthma, and epigenome-wide studies have shown variations in DNA methylation in many genes involved in asthma [60] along with fetal lung tissue and placental DNA methylation [61]. Nicotine exposure results in the generational transmission of asthma, showing epigenetic changes and DNA methylation, and H3 and H4 acetylation [62]. A 2017 study has highlighted the three CpG sites of methylation by NO_2_ from cord blood samples and a dose-dependent NO_2_ connection with pregnancy [63]. Mixtures of gases and particulate matter are the main components of air pollution. Air pollution induces DNA methylation from an early age to old age [64]. Research has reported hypomethylation of *iNOS* by particulate matter 2.5 (PM2.5) and particulate matter 10 (PM10) [65], methylation of *ACSL3* by traffic-related air pollution (TRAP) [66], and hypermethylation of IL-4 and IFN-*γ* CpG sites by exhaust particulate matter (DEP) [67]. DEP is associated with increased IgE levels in asthma [67]. Together, prenatal nicotine exposure, air pollution, PM2.5, and DEP induce epigenetic changes in genes through DNA methylation, resulting in distinct asthma phenotypes.

A study in 2019 showed that lead and cadmium are associated with asthma and other allergic conditions, and an interestingly significant degree of airflow obstruction is associated with the serum level of these metals [68]. Vanadium induces DNA methylation in air pollution-related asthma [69]. Occupational pesticide exposure and differential DNA methylation are correlated with symptomatic airflow obstruction [70].

Environmental microbial agents play an immune-modulating role under many conditions. The clinical asthma characteristics and airway microbiota have shown a significant correlation between asthma control and various pathogens [71]. Rhinovirus induces IL-33-dependent type 2 asthma exacerbations [72], and rhinovirus-induced DNA methylation changes on many genes are linked with asthma, especially the substantial changes seen on gene *SMAD3* [73].

Ovalbumin (OVA) and house dust mites (HDM) are the most common allergen used in the mouse model of allergic asthma for the induction of the Th2 pathway. Other asthma models use paramyxovirus, *Chlamydia muridarum*, Rotavirus 1 (RV1), and *Haemophilus influenzae* for the induction of non-Th2 inflammation. HDM exposure has been found to induce DNA methylation of various potentially important genes for allergic asthma development [74]. In allergic rhinitis, a close entity in asthma, DNA hypomethylation of the IL-13 gene was observed after HDM sensitization [75]. DNA methylation changes in several genes (*FOXO1*, *RUNX1*, *SP1*, and *APP*) are potentially crucial for the development of asthma in the OVA model [76].

The role of diet, folate, vitamin B12, vitamin C, calcium, vitamin D, fish oil, and antibiotics in pregnancy and asthma has been established [77–80]. The association between dietary intake and childhood asthma has identified that dietary intake contributes to DNA methylation [77]. The nutrients involved in the one-carbon metabolism pathway (selenium and several others) are associated with improved asthma status, but some dietary constituents are associated with both extensive and gene-specific methylation in pediatric asthma [77]. Novel epigenetic loci associated with folate and vitamin B12 have been noted in a large-scale epigenome-wide study in 2019, and a negative association between folate and DNA methylation was found [78]. These data show that dietary intake and some nutrients and vitamins are responsible for methylation changes in asthma-related genes and result in different phenotypes as well as remission of asthma.

## 4. Histone Modifications

Histones are a highly alkaline principal fundamental protein component of chromatin that acts as a spool for DNA winding. The unwound DNA length to width ratio would be beyond 10 million to 1. Therefore, it is considered a crucial element for genetic stability, packaging, and gene expression.

Histone modification, an epigenetic mechanism, is an influencing factor in the pathogenesis and development of asthma, affecting the maturation and differentiation of cells involved in asthma [81] (Figure 2 and Table 2). Histone modification usually occurs at the N-terminal with possible modifications on each “basic” residue, but common residue targets for modifications are lysine, serine, arginine, and tyrosine threonine. Acetylation, methylation, phosphorylation, ubiquitination, and sumoylation are well-known histone modification mechanisms. Histone acetylation and histone methylation are the most studied and are better documented [82].

Allfrey et al. have reported the first documented case of histone acetylation in 1964 from calf thymus [83], and the link between chromatin and histone acetylation was published in 1988 [84]. Histone acetyltransferases (HATs) and histone deacetylases (HDACs) function in opposition to each other as acetylation by HATs favors gene expression and deacetylation by HDACs is responsible for gene silencing. The imbalance of HDACs and HATs is the basics for impaired gene expression and a factor that contributes to asthma [16]. The subgroups of HATs and HDACs are beyond the scope of this review and are not explained here. The addition of mono-, di-, or trimethyl groups to lysine or arginine residues of histone tails favors histone methylation through the mediator histone methyltransferases (HMTs), while histone demethylases (HDMs) oppose HMTs. Enzymes of HMTs (“writers”) and HDMs (“erasers”) collectively balance a dynamic and static histone methyl landscape.

The addition of phosphate, phosphorylation, is mediated by kinases, while phosphatases remove phosphate. Similarly, histone ubiquitination is mediated by ubiquitin ligases and opposed by ubiquitin-specific peptidases, also known as deubiquitinating enzymes. Histone sumoylation is mediated by the histone sumoylation proteins, also known as the small ubiquitin-like modifier (SUMO) protein. Interestingly, although the biological process of DNA methylation and histone methylation differs, they mutually interact with each other [85].

### 4.1. Histone Modifications and Asthma

HDAC2 reduction signifies the possibility that the poor effectiveness of corticosteroids and inflammatory genes may be regulated by deacetylation of the glucocorticoid receptor through HDAC2 [87]. Activation of the *Notch* gene is crucial for Th2 response in asthma, and *Notch 1* dysregulated signaling of T cells is observed through the hyperacetylation of H3K9, H3K14, H3K18, H3K27, and H3K16 and trimethylation of H3K4 and H3K79 [88]. Suppression of *Notch1* expression by HAT inhibitors reduced Th2 cytokines, especially IL-4, IL-5, and IL-13 [88], and it may provide a therapeutic alternative for asthma. *LAT* is a protein-coding gene of T cells, and its deficiency enhances Th2 proliferation. *LAT* hypoacetylation results in the inhibition of *LAT* in an allergic asthma model [89]. Self-renewal and reprogramming of somatic cells are mediated by *SOX2*, and *HDAC1* mediates the remodeling of the asthmatic epithelium, while its inhibition prevents airway remodeling through *SOX2* [90].

Increased expression levels of *IFN-γ*, *IL-4*, *IL-17A*, *IL-17F*, and transcriptional factors of Th17 and Treg cells (*RORγt* and *Foxp3*) have been observed with H3K4 trimethylation (H3K4me3) [91]. *Foxp3* and *IL-13* gene acetylation is associated with childhood asthma [92]. Histone hyperacetylation of *ORMDL3* is associated with asthma [93], and increased *ORMDL3* expression is associated with airway remodeling and Ca^2+^ oscillations. *EGFR* plays an important role in the growth and maintenance of airway epithelium. *Delta Np63* induces epithelial shedding, and *STAT6* is involved in chronic inflammation of the airway mucosa. TSSs of *EGFR*, *STAT6*, and *Delta Np63* show increased acetylation of H3K18 in the airway epithelium of asthmatics [94].

Chemokine ligand-8, a neutrophil activator produced by macrophages, and H3K18 acetylation leads to increased secretion of this activator from airway smooth muscles [95]. CCR4 is a mediator for Th2 cell recruitment, and CCL5 is a leukocyte chemoattractant found to be high in asthma. *CCR4* and *CCL5* dimethylation (H3K4me2) at single nucleotide polymorphisms is associated with Th2 differentiation [96].

### 4.2. Environmental Exposures, Histone Modifications, and Asthma Risk

Studies have documented environment-induced effects on histone modification and asthma risk. Smoking induces increased acetylation at H3 and H4, and transmission of asthma in subsequent generations has been studied in an animal model [62, 97]. HDAC2 downregulation results in the upregulation of IL-8, TNF, and GM-CSF and glucocorticoid inhibition in smokers [98]. A study on maternal E-cigarette exposure has also showed epigenetic and cognitive changes in offspring [99]. Interestingly, a multigeneration analysis of the Respiratory Health study showed that the father's environmental exposures before conception can influence offspring's respiratory health in later life [100]. There is stronger asthma morbidity in children than adolescents [101], and histone H3 modification at birth is associated with prenatal air pollution, which is a risk factor for later-life air pollution-associated diseases [102]. The association of traffic-derived particulate matter, mainly black carbon, and global histone H3 modification has been established [103].

Environmental microbes may play roles in the immune response's modulation of immune responses. Histone H4 acetylation of essential Th2 genes is modified by nematode infections, such as ascariasis and HDM [104]. Interestingly, *Acinetobacter lwoffii F78* administration to experimental mice during the prenatal period has shown beneficial effects and prevented the asthma phenotype. In this case, H4 acetylation of *IFN-γ* and deacetylation of *IL-4* promoter genes result in the downregulation of Th2 cytokines, showing transmaternal asthma protection [105]. Gut microbiota inhibits HDACs, resulting in Th1/Th17 effector cell polarization along with global hyperacetylation of intestinal macrophages, which produce butyrate and short-chain fatty acids that inhibit the proliferation and differentiation of Th2 cytokines in mice [106, 107]. Interestingly, in our OVA asthma model, alanylglutamine reduced cytokine production and relieved asthma symptoms by regulating gut microbiota composition [108].

Nickel, a silver-colored metal found in a large amount in some grains, vegetables, and fruits, can cause allergies in susceptible people and are associated with asthma and wheezing [109]. Nickel-induced posttranscriptional modification continues even after exposure through an epigenetic mechanism [110], while nickel exposure with hypoxic insult enhances H3K9me2 and H3K9me3 by inhibiting HDMs [111]. Interestingly, it can induce histone ubiquitination. Folate is an essential component for the repair and synthesis of DNA and other genetic materials. GATA3 (H3/H4) and IL-9 (H4) acetylation was noted with very high maternal folate levels in CD4+ T cells from cord blood [112]. GATA3 is a transcription factor for Th2 cells, and IL-9 is produced by Th9 and Th2 cells. High folate levels during pregnancy indicate the possibility of enhancement of Th2 promoter genes [112].

## 5. miRNAs (Noncoding RNA)

In 1993, Victor Ambros' laboratory discovered miRNAs and simultaneously identified the miRNA target gene. miRNAs are approximately 22-25 nucleotide single-stranded small noncoding RNA molecules transcribed from the DNA; however, they are still not translated into proteins and have a role in gene expression either by blocking or by altering mRNA translation stability [86]. miRNAs bind to the 3′ untranslated region of the gene with complementary sequence interaction and repress its expression.

### 5.1. miRNAs and Asthma

Although the discovery of miRNA is approaching thirty years, its role in asthma became a topic of investigation in the last decade. Many mouse and human studies have been conducted to identify the role of microRNAs via epigenetic changes in genes, and altered miRNA expression has been found in various lung diseases, including asthma [113, 114] (Figure 2 and Table 3). In the following paragraphs, we review the epigenetic changes in asthma genes through miRNAs, resulting in various phenotypic presentations and remission of asthma along with the possibility of diagnostic biomarkers and therapeutic potential from different studies.

A recent study published in 2020 has shown that circulatory miRNA levels correlate with the clinical status in patients with allergic and nonallergic asthma [115], and circulatory miRNA expression with T cell cytokines characterizes asthma exacerbation [116]. Some studies have even postulated that miRNAs can be considered a noninvasive biomarker in the diagnosis and identification of allergic asthma [117].

miR-21 prevents the expression of IL-3, IL-5, and IL-12, while the removal of miR-21 induces the production of IFN-*γ* and IL-12 from dendritic cells with reduced IFN-*γ* production from CD4+ T cells [118]. miR-21 controls type 1/type 2 balance in steroid-insensitive, OVA, and HDM model type 2 high asthma and is highly expressed in asthmatic children, with an inverse association between IL-p35 and miR-21 [118–120]. A study published in 2020 has shown that LncRNA-CASC7 enhances corticosteroid sensitivity through miR-21 by inhibiting the PI3K/AKT pathway and highlighted a potential therapeutic option in severe asthma, and it may also serve as a predictor of inhaled corticosteroid treatment response over time in asthma [141]. Th2 cytokines, especially IL-5 and IL-13, are decreased by miR-146a on ILC2 by inhibiting IRAK1 [121], and miR-146a also alters neutrophil migration from bronchial epithelial cells in asthma [122]. Similarly, the let-7 family of miRNAs found exclusively in the asthmatic lungs modulates the IL-13 expression, and its inhibition inhibited the production of allergic cytokines [123]. Together, these studies suggest the role of miRNAs in type 1/type 2, steroid-insensitive asthma and guide the prediction of asthma and the possibility of future therapy.

The increased expression level of miR-1248 is believed to be involved in the pathogenesis of asthma. miR-1248 increases IL-5 expression by interacting with the 3′UTR of the gene [124]. miR-126 promotes Th2 eosinophilic asthma, and antagomir-induced inhibition of miR-126 suppresses eosinophil recruitment [125]. Upregulated miR-126 in pediatric asthma correlates with immune imbalance and is postulated as a possible serum marker in the diagnosis and management of asthma [142]. miR-155 is the first identified and most studied miRNA to be linked to various diseases, including allergy and asthma. A Th2 association is seen with miR-221 and miR-155 along with other cells involved in allergic responses, such as mast cells [143], eosinophils [126], and macrophages. However, it has recently been found that airway mir-155 also signifies Th1 cytokine polarization with viral respiratory infections [127]. Similarly, a study in 2020 explained that miR-1 is a direct inhibitor of eosinophilic response in asthma and chronic rhinosinusitis [128]. The miR-23-27-24 cluster controls T cell functions and differentiation, and miR-24 and miR-27 inhibit Th2 differentiation, resulting in inhibited IL-4 [130]. These observations signify the role of miRNAs in allergic, eosinophilic, and virus exacerbated asthma, as well as serum biomarkers in the diagnosis and prediction of pediatric asthma.

In asthma, miR-16 and lung function parameters are negatively correlated, and inhaled *β2*-agonist responses are altered by miR-16 through *ADRB2* [131]. Circular RNA (circHIPK3) is involved in the proliferation of smooth muscles and airway remodeling in asthma through the miR-326/STIM1 pathway [132]. Pulmonary macrophage polarization is mediated by miR-130a-3p and miR-142-5p and is associated with airway remodeling [144]. The Th2 cytokine level increases with increased airway expression of miR-19a through PTEN and deubiquitinase A20 [133], and exaggerated remodeling of the airway is noted with reduced miR-19a [134]. VEGF-A is overexpressed in sputum and serum of asthmatic patients, and lower expression of has-miR-15a is related to the increased expression of VEGF-A in CD4+ T cells [135]. Airway inflammation and remodeling are reduced by miR-19b through TSLP mediated *STAT3* inhibition [136] and *MMP-16* and *ATG7* by miR-192-5p [137]. This mechanism may provide a therapeutic target for airway remodeling of asthma. Downregulation of miR-1 in the endothelium results in Th2 inflammation via VEGF, with increased expression of myeloproliferative leukemia virus oncogene [129]. Thus, research has shown the role of multiple miRNAs in airway inflammation and remodeling.

miR-27-3p is an important player in experimental pediatric asthma that mediates immune reactions. *SYK* and *EGFR* genes are targets of miR-27-3p that influence the PI3K-AKT pathway and cytokine production [138]. An inverse correlation between IL-22 and expression of miR-323-3p has shown enhanced expression of miR-323-3p in IL-22 and IL-17 expressing cells with decreased IL-22 production and TGF-*β* suppression [139]. hsa-miR-20a-5p induces allergic inflammation by preventing HDAC4 expression and epigenetically upregulating IL-10 in activated human mast cell 1 [140].

Taken together, these studies have shown the role of multiple miRNAs in Th1/Th2 polarization, disease presentation, correlation of clinical status, severity, remission, noninvasive biomarker, therapeutic potential, and response to therapy in asthma.

### 5.2. Environmental Exposures, miRNAs, and Asthma Risk

Some *in vivo* and *in vitro* studies have identified an association between environmental factors, miRNAs, and asthma. Different types of allergens, such as cigarette smoking, air pollution, agriculture pesticides, metals, and various microbes, induce epigenetic changes in genes through the miRNA and show various phenotypic presentations and even remission of asthma. This section reviews the association between environmental factors, miRNAs, and asthma. However, some of the associations have already been described in miRNAs and Asthma.

OVA induces experimental asthma by downregulating let-7 miRNAs and IL-13 expression [123]. Exogenous administration of let-7 miRNA results in reduced eosinophil recruitment [123]. HDM is an important triggering factor for asthma and is responsible for lung-specific miRNA expression. HDM triggers IL-33 release, which activates and proliferates ILC2, which ultimately releases Th2 cytokines, fulfilling the clinical parameter of allergic asthma. miR-155 knockout HDM sensitized mice displaced low levels of IL-33 and ILC2, suggesting that HDM induces asthma through the critical role of miR-155 [145]. An allergen mix, containing dust mite, ragweed, and aspergillus (DRA allergen), reduces the miR-451 levels in pulmonary macrophages, and depletion of miR-451 results in asthma through the expression of sirtuin 2 [146]. Interestingly, IgE is involved in the pathogenesis of asthma-related hypotension through *NCX1* downregulation and miR-212-5p activation, as shown by the significant restoration of blood pressure after the knockdown of miR-212-5p in asthmatic mice [147]. OVA, HDM, and DRA allergen are involved in the pathogenesis of asthma through the epigenetic changes in miRNAs.

miR-335-5p inhibits inflammatory cytokines, and cigarette smoking lowers the expression of miR-335-5p in parenchymal lung fibroblast [148]. The upregulation of miR-500 and miR-181 and downregulation of miR-128b and miR-218 are seen in the airway epithelium of smokers [149], suggesting a possible association with cigarette smoke. Environmental tobacco smoke (ETS) during pregnancy promotes epigenetic changes and transgenerational transmission of allergic asthma with increased miR-221 and miR-16 levels and reduced levels of miR-130a [131, 150]. Maternal smoking increases AXL methylation and reduces miR-199a expression, resulting in the alteration of childhood respiratory symptoms [151]. Together, these studies have shown an association between smoking and miRNAs in childhood asthma.

Recent findings have shown that indoor air pollution changes serum miR-155 levels and aggravates asthma [152]. As an air pollutant, ozone increases the expression of miR-132, miR-143, miR-145, miR-199a, miR-199b-5p, miR-222, miR-223, miR-25, miR-424, and miR-582-5p in human airways [153]. CD4+ T cell-derived Th1/Th2 imbalance is an initiating factor in ozone exacerbated asthma through PVT1-miR-15a-5p/miR-29c-3p signaling [154], and miR-15b-5p is considered a biomarker for identifying patients with asthma chronic obstructive pulmonary disease overlap [155]. PM2.5 promotes asthma by expressing miR-206 in lung tissue of asthmatic mice by inhibiting SOD1 expression and ultimately increasing the reactive oxygen species level [156]. miR-224 plays an inhibitory role in PM2.5-induced asthma by inhibiting Th17 and TLR2, resulting in reduced secretion of IL-17, IL-4, and IL-5 [157]. Resveratrol, a natural phenol compound commonly found in peanuts, wine, berries, and red grapes, suppresses the asthma-associated immune response mediated by the upregulation of *FOXP3* through downregulation of miR-34a [158]. Indoor air pollution, ozone, PM2.5, and some other natural compounds affect the expression of multiple miRNAs through epigenetic changes in asthma.

The association between serum levels of heavy metals, such as cadmium, mercury, and lead, and asthma and allergic rhinitis has been established. miR-148a, miR-211, miR-520c-3p, and miR-572 are associated with lead poisoning and are biomarkers of lead susceptibility in the Chinese worker population [159]. miR-211 inhibits the TGF-*β* pathway via inhibin-*β* A [160]. Cadmium downregulates miR-30 through the upregulation of the transcriptional factor SNAIL and can be considered an important step in some lung diseases [161]. miRNA-30a-3p reduces eosinophil activity in asthma by targeting CCR3 [150, 161]. Occupational exposure to mercury upregulated miR-92a-3p and miR-486-5p in females working in a mercury thermometer factory [162]. These studies have shown an association between environmental occupational exposure in asthma and the expression of multiple miRNAs.

The influenza virus, rhinovirus, and respiratory syncytial virus (RSV) cause respiratory illness and exacerbate asthma [71, 72, 74]. Increased miR-155 in children with *in vivo* viral respiratory infection has shown increased IFN-*γ* production, Th1 polarization (IFN-*γ*/IL-4 ratio), and proinflammatory responses [127]. Rhinovirus-infected severe asthmatic alveolar macrophage exhibits reduced TLR7 expression levels due to miR-150, miR-152, and miR-375 [163]. Reduced hsa-miR-34b/c-5p from RSV-infected human bronchial epithelial cells results in mucus secretion (MUC5AC secretion) through the AP-1 pathway [164]. In severe asthma, miR-22 expression, along with its targets CD147 and HDAC4, is dysregulated by the influenza virus [165], which may explain the H1N1 influenza infection-induced airway remodeling in severe asthma [165]. These viruses are involved in the pathogenesis, airway remodeling, and Th1 polarization of asthma through the epigenetic expression of various miRNAs.

## 6. Is There an Epigenetic Link between Childhood Asthma and Adult-Onset/Old Age Asthma?

Asthma predominantly occurring during childhood is childhood asthma, while asthma occurring during adulthood and old age is termed adult-onset/old age asthma. The T2 phenotypes of asthma (T2 high) include early-onset allergic and late-onset nonallergic eosinophilic asthma. The common Th2 biomarkers used in clinical practice are mainly blood eosinophils, FeNO, and IgE levels. Interestingly, the majority of people with T2 asthma respond well to standard therapy with inhaled corticosteroids [4], whereas non-T2 (T2 low) asthma is still obscure, is a neutrophilic and paucigranulocytic heterogenous type predominant with adult-onset, is associated with corticosteroid resistance (less responsive), and is inflammation-driven through IL-17, IL-6, and IL-23 with airway smooth muscle or neural dysfunction and may be associated with comorbidities like obesity and gastroesophageal reflux disease [5, 6].

However, we should not forget that these profiles are not strictly age-limited (children and adults may have eosinophilic, neutrophilic, or even paucigranulocytic asthma with overlapping steroid response), and specific and useful signature biomarkers are lacking for non-T2 asthma apart from the absence of T2 high inflammation. Together, these observations suggest the two phases of asthma in human life as childhood asthma and adult-onset/old age asthma.

Environmental factors are asthma epigenetic influencers that induce epigenetic changes in genes through DNA methylation, histone modifications, and miRNAs, as reviewed in Sections 3.2, 4.2, and 5.2, respectively. Some environmental factors affect childhood asthma, while others are involved in the pathogenesis or remission of adult-onset/old age asthma through epigenetic changes; however, some overlap still exists in both.  Table 4 shows the environmental exposures and the respiratory outcomes relating to childhood and adult-onset/old age asthma, including a study from an animal asthma model.

Maternal tobacco smoking, *in utero* nicotine exposure, maternal E-cigarette exposure, and even secondhand smoke (ETS) during pregnancy are associated with increased risk and rate of childhood asthma, elevated IgE levels, wheezing, alteration of childhood respiratory symptoms, and increased bronchial activity [57, 97, 120, 148, 149]. Prenatal air pollution is a risk factor for childhood asthma and a risk factor for later-life air pollution-related diseases [100]. Similarly, indoor air pollution aggravates childhood asthma [150]. Viral respiratory infections in children exacerbate asthma and increase Th1 polarization, while paramyxovirus, *Chlamydia muridarum*, Rotavirus 1, and *Haemophilus influenzae* induce non-Th2 inflammation [69, 71, 72, 129]. Nutrients, such as folate (methyl group donors), vitamin B12, vitamin D, and selenium (one-carbon metabolism pathway), play important roles in childhood asthma pathogenesis. There are many conflicting studies on childhood asthma risk with maternal folate levels. One study has shown that high folate levels during pregnancy are associated with the possibility of enhanced Th2 promoter genes [110]. Interestingly, *Acinetobacter lwoffii F78* prenatal administration prevents childhood asthma in an animal model [103]. Together, these environmental exposures induce epigenetic changes and play a role in the pathogenesis or remission of childhood asthma.

Multiple environmental factors are also associated with adult-onset/old age asthma. Occupational exposure to lead, cadmium, mercury, nickel, and pesticides results in asthma and symptomatic airflow obstruction in adults [66, 68]. Rhinovirus and ascariasis are responsible for type 2 asthma and exacerbations in adults [70, 102]. Genetic predisposition (asthmatic parents), mode of delivery, obesity, hormonal level, and hygiene are also related to childhood asthma as well as adult-onset/old age asthma.

Overall, human asthma studies and experimental asthma models have shown the involvement of various environmental factors in the pathogenesis of childhood and adult-onset/old age asthma [65, 103–106, 140, 152, 154] by inducing epigenetic changes through the genes (Sections 3.2, 4.2, and 5.2) and resulting in various phenotypic presentations of childhood and adult-onset/old age asthma.

**Figure 3:**
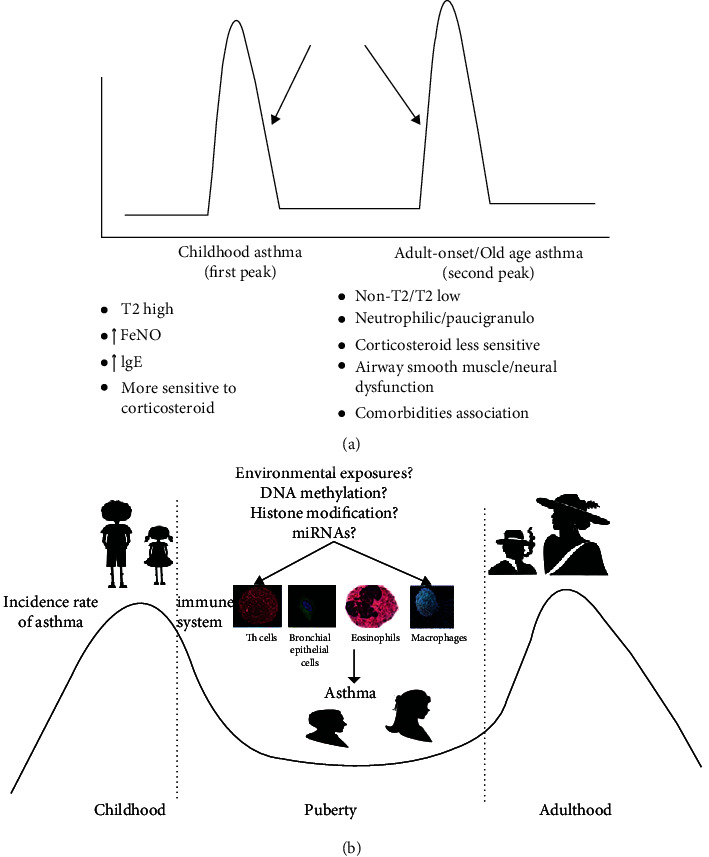
The two peaks of human asthma and the role of epigenetics in the cellular transformation of childhood asthma to adult-onset/old age asthma. (a) The two peaks of human asthma are childhood asthma (↑ IgE, ↑ eosinophils, FeNO, corticosteroid sensitive, and T2 high) and adult-onset/old age asthma (neutrophilic/paucigranulocytic, corticosteroid less sensitive, and non-T2 asthma) with airway smooth muscle or neural dysfunction, possible association with comorbidities [5, 6], and periods of remission in some cases. However, we should not forget that these profiles are not strictly age-limited (children and adults may have eosinophilic, neutrophilic, or even paucigranulocytic asthma with overlapping steroid response), and several other components of the immune, neuronal, and hormonal responses are also involved with asthma. (b) The two peaks of asthma (childhood asthma and adulthood as adult-onset/old age asthma). In general, there is male dominance in childhood asthma, with a shift to female dominance in later peak. Components of epigenetic mechanisms, as well as cells involved in asthma, are also shown, as their interaction is needed in asthma epigenetics. The long arrows in (a) show the possible point of commencement and involvement of epigenetic mechanisms, as we believe, in the cellular transformation of childhood asthma to adult-onset/old age asthma, although further epigenetic research is required in the future. IgE: immunoglobulin E; miRNAs: micro-RNAs; FeNO: fractional exhaled nitric oxide.

Is there a link between childhood asthma and adult-onset/old age asthma? Does adult-onset/old age asthma represent the persistence or relapse of childhood asthma? How does increased IgE, eosinophil-predominant, and steroid-sensitive T2 asthma change into neutrophilic/paucigranulocytic, steroid-insensitive non-T2 severe asthma with age? At this point, through the review of components of epigenetic mechanisms (DNA methylation, histone modifications, and miRNAs) and genetics and environmental factors, we strongly believe that the epigenetic mechanisms are involved in the cellular transformation of childhood asthma to adult-onset/old age asthma (see Figures 3(a) and 3(b)), although further research is required. Further molecular- and genetic-level asthma epigenetic studies are needed to clarify the occurrence of cellular transformation of childhood asthma to a different entity of adult-onset/old age asthma (persistence, relapse, and new-onset adulthood/old age asthma).

## 7. Conclusion and Perspectives

Asthma is a mysterious disease with heterogeneity in etiology, pathogenesis, and clinical phenotypes under genetic influences. Asthma heritability is established through genetic studies, and environmental factors are also influential in asthma. However, genetic and environmental factors cannot entirely explain the asthma endotypes and phenotypes.

Emerging evidence suggests that epigenetic mechanisms link genetic and environmental factors with asthma trajectories. Epigenetics is defined as the study of heritable phenotypic changes due to activation, inhibition, or repression of genes without changes in the underlying DNA environment. The most studied epigenetic asthma phenomena are DNA methylation, histone modifications, and small noncoding RNA (miRNAs).

Asthma epigenetics play an important role in immune response and upregulation of various cellular functions, including T cell differentiation, Th cell balance, changes in the expression of inflammatory genes resulting in asthma, and remission or protection in some cases. Asthma epigenetic studies can be a valuable approach to identify the increasing incidence of asthma and equally crucial for the study of environmental factors involved in asthma pathogenesis. Although more clinical, epidemiological, environmental, cellular, and interventional *in vivo*, *in vitro*, and human studies are needed, we believe that the asthma epigenetic mechanism can be a novel process for the identification of unsolved pathogenesis, new asthmatic loci, heterogeneity, and treatment options through the discovery of new drugs. Identification of epigenetic markers is vital for identifying asthma endotypes, phenotypes, personalized treatments, and prevention. In the future, we expect that the study of asthma epigenetics will guide us towards gene therapy, T cell therapy, and even provide directions for treating severe uncontrolled drug-insensitive asthma and predict long-term outcomes.

Given the queries raised, we believe that the epigenetic mechanisms play vital roles in the cellular transformation of childhood asthma (eosinophil-predominant, steroid-sensitive T2 asthma) to adult-onset/old age asthma (neutrophilic/paucigranulocytic, steroid less-sensitive non-T2 severe asthma). However, we expect further epigenetic studies on this aspect in the future.

Although epigenetic studies of asthma are still at early stages, we hope that the mystery surrounding asthma will be solved to some extent via the integration of omics data via novel findings for improved phenotyping, diagnoses, and asthma treatments, as well as the mechanism that will eventually prevent its development. Together, in view of all asthma epigenetic studies, we can conclude that asthma epigenetics are a valuable, novel approach to solve the mysteries surrounding asthma and will guide us to the discovery of new therapeutic agents and prediction of long-term outcomes in severe cases and aid in the understanding of the cellular transformation of childhood asthma to adult-onset/old age asthma.

## Figures and Tables

**Table 1:** Some DNA methylated genes with genetic loci, epigenetic modifications, and clinical outcome from different tissues/samples.

Tissues/samples	Genes	Genetic location	Epigenetic modification	Clinical outcome	References
Nasal epithelium	*ALOX15*, *POSTN*	17p13.2, 13q13.3	Hypomethylation	Th2 response, childhood asthma	[30]
Blood	*ALOX12*	17p13.1	Hypomethylation	Childhood persistent wheezing	[34]
Blood	*IFN-γ*, *FOXP3*	12q15, Xp11.23	Hypermethylation	Impaired T cell function, Treg and T effector repression	[35]
Blood	*IL-13*, *RUNX3*, and *TIGIT*	5q31.1, 1p36.11, and 3q13.31	Hypomethylation	Childhood asthma	[36]
Cord blood	*IL-2 Site1*	4q27	Hypermethylation	Severe asthma in children	[37]
Blood	IL-5RA	3p26.2	Hypomethylation	Asthma (in teens)	[38]
Cord blood	*GATA3*	10p14	Hypermethylation	Reduced asthma risk	[40]
HapMap LCLs	*ZPBP2*	17q12-q21	Hypomethylation	Asthma	[41]
Blood	*CYP26A1*	10q23.33	Hypermethylation	Aspirin intolerant asthma and allergy	[42]
Nasal epithelium	*TET1*	10q21.3	Hypomethylation	Asthma	[43]
Blood	*IL1R2*	2q11.2	Hypermethylation	Asthma	[45]
Newborn blood spots	*AXL*	19q13.2	Hypermethylation	Wheezing (girls > boys)	[46]
Buccal cells	*ARG2*	14q24.1	Hypermethylation	Decreased FeNO in children with asthma	[47]
Airway epithelium	*IL-6*, *NOS2*	7p15.3, 17q11.2	Hypomethylation	Increased FeNO, childhood asthma	[48]
Blood	*ADRB2*	5q31-33	Hypermethylation	Severe childhood asthma	[49]
Saliva/blood	*ADRB2*	5q31-33	Hypomethylation	Reduced dyspnea in asthmatic children	[50]
Sputum	*PCDH20*	13q21.2	Hypermethylation	Asthma in adults	[51]
Nasal epithelium	*CDH26*, *CDHR3*	20q13.33, 7q22.3	Hypomethylation	Differentiate atopy with atopic asthma	[52]
Cord blood	*C7orf50*, *ZAR1*	7p22.3, 4p11	Hypomethylation	Associated with IgE levels	[53]
Airway epithelium	*STAT5A*	17q21.2	Hypermethylation	Enhanced Th1 response, decreased EOS recruitment	[54]
Blood	*WNT2*	7q31.2	Hypermethylation	Neutrophilic asthma	[55]
Endobronchial AEC	*ORMDL3*	17q12-21	Hypermethylation	Asthma in adults	[56]
Lung AEC	IL-13	5q31	Hypermethylation	Asthma	[57]

For genetic loci: https://www.ncbi.nlm.nih.gov/gene/ or https://www.genecards.org/ or cited references.

**Table 2:** Some histone-modified genes, epigenetic modifications with clinical outcome.

Genes	Epigenetic modifications	Clinical outcome	References
Glucocorticoid receptor (*GRa*)	HDAC2 downregulation	Severe asthma	[87]
*Notch-1*	Hyperacetylation of H3K9, H3K14, H3K18, H3K27, and H3K16 and trimethylation of H3K4 and H3K79	Asthma	[88]
*LAT*	Hypoacetylation	Asthma	[89]
SOX2	HDAC1 upregulation	Asthma	[90]
*IFN-γ*, *IL-17A*, *IL-17F*, *IL-4*, *Foxp3*, *RORγt*	H3K4 trimethylation (H3K4me3)	Asthma/allergic diseases	[91]
*IL-13*, *Foxp3*	Acetylation	Asthma	[92]
*ORMDL3*	Hyperacetylation	Asthma	[93]
*ΔNp63*, *STAT6*, *EGFR*	H3K18 acetylation	Asthma	[94]
*CXCL8*	H3K18 acetylation	Asthma	[95]
*CCR4*, *CCL5*	H3K4 dimethylation	Asthma	[96]

**Table 3:** miRNAs, epigenetic modifications, and clinical outcome.

Genes	Epigenetic modifications	Clinical outcomes	References
*IL-3*, *IL-5*, and *IL-12*	miR-21	Asthma	[118]
*IL-12p35*	miR-21	Severe asthma	[118–120]
*IRAK1*	miR-146a	Neutrophil migration, IL-5, IL-33 expression	[121, 122]
*IL-13*	Let-7	Asthma	[123]
*IL-5*	miR-1248	Asthma	[124]
*TOM1*	miR-126	Asthma/eosinophil recruitment	[125]
*S1pr1*	miR-155	Th2/Th1 response	[126, 127]
*VEGF/Mpl*, *SELP*, *CCL26*, *TSLP*	miR-1	Th2 inflammation/eosinophil regulation	[128, 129]
*IL-4 regulator genes*	miR-23, miR-27	IL-4 expression	[130]
*ADRB2*	miR-16	Asthma	[131]
*miR-326/STIM1 axis*	circHIPK3	Asthmatic airway remodeling	[132]
*PTEN/A20*	miR-19a	Asthma and airway remodeling	[133, 134]
*VEGF*	miR-15a	Asthma	[135]
*TSLP*	miR-19b	Asthma/airway remodeling	[136]
*MMP-16*, *ATG7*	miR-192-5p	Airway remodeling	[137]
*SYK*, *EGFR*	miR-27-b-3p	Pediatric asthma	[138]
*IL-22*	miR-323-3p	Asthma	[139]
*HDAC4*	miR-20a-5p	Allergic inflammation/asthma	[140]

**Table 4:** Environmental exposures and respiratory outcomes in asthma.

Environmental exposures	Outcomes
Prenatal and early childhood maternal tobacco smoking	Risk of childhood asthma [57], alteration of childhood respiratory symptoms [149]
Nicotine exposure (*in utero*)	Transgenerational transmission of asthma (animal model) [60, 95]
Maternal E-cigarette exposure	Offspring epigenetic and cognitive changes [97]
Second-hand smoke during pregnancy (ETS)	Transgenerational transmission of allergic asthma [120, 148]
Father's environmental exposures before conception	Influence offspring's respiratory health [98]
Diesel exhaust particulate (DEP) matter	Increased IgE levels in asthma (animal model) [65]
Lead, cadmium, and mercury	Asthma in adults, airflow obstruction [66]
Vanadium	Induces DNA methylation on air pollution-related asthma in children [67]
Occupational pesticide exposure	Symptomatic airflow obstruction in adults [68]
Air pollution	DNA methylation from early life to old age, childhood asthma [62, 99, 100]
Nickel exposure	Asthma and wheezing in susceptible adults [107, 108]
Indoor air pollution	Aggravates asthma in children [150]
Ozone	Ozone exacerbated asthma, Th1/Th2 imbalance (animal model) [152]
Particulate matter (PM2.5)	Promotes asthma (animal model) [154]
Viral respiratory infection in children	Asthma exacerbations, increased Th1 polarization, non-T2 inflammation [69, 71, 72, 129]
Rhinovirus	IL-33-dependent type 2 asthma exacerbations in adults [70]
Ascariasis	Th2 asthma in adults [102]
Acinetobacter lwoffii F78 prenatal administration	Prevention from childhood asthma (animal model) [103]
Gut microbiota	Inhibit differentiation of Th2 cytokines (animal model) [104–106]
OVA, HDM	T2 asthma (animal model) [73, 74]
Dust mite, ragweed, and aspergillus (DRA allergen)	Th2 mediated asthma (animal model) [140]
High folate during pregnancy	Possibility of enhancement of Th2 promoter genes [110]

## Abbreviations

CpG: Cytosine-phosphate-guanine
DNMT: DNA methyltransferase
HAT: Histone acetyltransferase
HDACs: Histone deacetylases
HDMs: Histone demethylases
HMT: Histone methyltransferase
TSS: Transcription start site.

## Additional Points

*Points to Remember*. (i) Genetic and environmental factors are unable to completely explain asthma pathogenesis and phenotypes. (ii) Asthma epigenetics has become a “hot topic” due to the search for alternative explanations for mysterious phenotypes and heterogeneity of asthma. (iii) DNA methylation, histone modifications, and miRNAs are the primary components of asthma epigenetics. (iv) Epigenetic mechanism links genetic and environmental factors with developmental trajectories and plays an important role in T cell differentiation, while influencing the Th cell balance, and epigenetic marks can be inherited to subsequent generations. (v) Asthma epigenetics may provide a novel mechanism for identifying the increased incidence, pathogenesis, and heterogeneity of asthma and a novel mechanism to identifying new asthmatic loci, associated environmental factors, and treatment options by the discovery of new drugs. (vi) In the future, asthma epigenetics will direct us to T cell therapy, gene therapy, and personalized asthma therapy and will give long-term predictability in severe cases. (vii) We believe that asthma epigenetics plays a vital role in the cellular transformation of childhood asthma to adult-onset/old age asthma. However, further research must be conducted in this field. (viii) Although the asthma epigenetic field is very promising and is still at early stages, we hope that the mystery surrounding asthma will be explained shortly and ultimately lead us to improved phenotyping, diagnosing, and treating of asthma.

## References

[1] November 2020, https://www.who.int/news-room/fact-sheets/detail/asthma

[2] November 2020, https://www.nhlbi.nih.gov/sites/default/files/media/docs/EPR3_Asthma_Full_Report_2007.pdf

[3] Sears M. R., Greene J. M., Willan A. R. (2003). A longitudinal, population-based, cohort study of childhood asthma followed to adulthood. *The New England Journal of Medicine*.

[4] Kaur R., Chupp G. (2019). Phenotypes and endotypes of adult asthma: moving toward precision medicine. *The Journal of Allergy and Clinical Immunology*.

[5] Diamant Z., Vijverberg S., Alving K. (2019). Toward clinically applicable biomarkers for asthma: an EAACI position paper. *Allergy*.

[6] Ray A., Kolls J. K. (2017). Neutrophilic inflammation in asthma and association with disease severity. *Trends in Immunology*.

[7] Akinbami L. J., Moorman J. E., Bailey C. (2012). Trends in asthma prevalence, health care use, and mortality in the United States, 2001-2010. *NCHS Data Brief*.

[8] GBD 2016 Disease and Injury Incidence and Prevalence Collaborators (2017). Global, regional, and national incidence, prevalence, and years lived with disability for 328 diseases and injuries for 195 countries, 1990-2016: a systematic analysis for the Global Burden of Disease Study 2016 [published correction appears in Lancet. 2017 Oct 28; 390 (10106): e38]. *The Lancet*.

[9] WHO|Disease burden and mortality estimates (2018). *Global health estimates 2016: deaths by cause, age, sex, by country and by region, 2000-2016*.

[10] McGeachie M. J., Stahl E. A., Himes B. E. (2013). Polygenic heritability estimates in pharmacogenetics: focus on asthma and related phenotypes. *Pharmacogenetics and Genomics*.

[11] Thomsen S. F., van der Sluis S., Kyvik K. O., Skytthe A., Backer V. (2010). Estimates of asthma heritability in a large twin sample. *Clinical and Experimental Allergy*.

[12] Edfors-Lubs M. L. (1971). Allergy in 7000 twin pairs. *Acta Allergologica*.

[13] Waddington C. (1942). Canalization of development and the inheritance of acquired characters. *Nature*.

[14] Nanney D. L. (1958). Epigenetic control systems. *Proceedings of the National Academy of Sciences of the United States of America*.

[15] Ptashne M. (2007). On the use of the word 'epigenetic'. *Current Biology*.

[16] Kabesch M., Adcock I. M. (2012). Epigenetics in asthma and COPD. *Biochimie*.

[17] McCarty M., Avery O. T. (1946). Studies on the chemical nature of the substance inducing transformation of pneumococcal TYPES. *The Journal of Experimental Medicine*.

[18] Moen E. L., Mariani C. J., Zullow H. (2015). New themes in the biological functions of 5-methylcytosine and 5-hydroxymethylcytosine. *Immunological Reviews*.

[19] Neidhart M., Neidhart M. (2016). Chapter 1- DNA methylation – introduction. *DNA Methylation and Complex Human Disease*.

[20] Ziller M. J., Gu H., Müller F. (2013). Charting a dynamic DNA methylation landscape of the human genome. *Nature*.

[21] Lamadema N., Burr S., Brewer A. C. (2019). Dynamic regulation of epigenetic demethylation by oxygen availability and cellular redox. *Free Radical Biology & Medicine*.

[22] Zemach A., McDaniel I. E., Silva P., Zilberman D. (2010). Genome-wide evolutionary analysis of eukaryotic DNA methylation. *Science*.

[23] Jones P. A. (2012). Functions of DNA methylation: islands, start sites, gene bodies and beyond. *Nature Reviews Genetics*.

[24] Jia A., Wang Y., Sun W. (2017). MBD2 regulates Th17 cell differentiation and experimental severe asthma by affecting IRF4 expression. *Mediators of Inflammation*.

[25] Sun W., Xiao B., Jia A. (2018). MBD2-mediated Th17 differentiation in severe asthma is associated with impaired SOCS3 expression. *Experimental Cell Research*.

[26] Oey H., Whitelaw E. (2014). On the meaning of the word ‘epimutation’. *Trends in Genetics*.

[27] Kurdyukov S., Bullock M. (2016). DNA methylation analysis: choosing the right method. *Biology*.

[28] Mondoulet L., Dioszeghy V., Busato F. (2019). Gata3 hypermethylation and Foxp3 hypomethylation are associated with sustained protection and bystander effect following epicutaneous immunotherapy in peanut-sensitized mice. *Allergy*.

[29] Xu C. J., Söderhäll C., Bustamante M. (2018). DNA methylation in childhood asthma: an epigenome-wide meta-analysis. *The Lancet Respiratory Medicine*.

[30] Yang I. V., Pedersen B. S., Liu A. H. (2017). The nasal methylome and childhood atopic asthma. *The Journal of Allergy and Clinical Immunology*.

[31] Chu H. W., Balzar S., Westcott J. Y. (2002). Expression and activation of 15-lipoxygenase pathway in severe asthma: relationship to eosinophilic phenotype and collagen deposition. *Clinical and Experimental Allergy*.

[32] Song Y. S., Yang E. M., Kim S. H., Jin H. J., Park H. S. (2012). Effect of genetic polymorphism of ALOX15 on aspirin-exacerbated respiratory disease. *International Archives of Allergy and Immunology*.

[33] Matsumoto H. (2020). Role of serum periostin in the management of asthma and its comorbidities. *Respiratory Investigation*.

[34] Morales E., Bustamante M., Vilahur N. (2012). DNA hypomethylation atALOX12Is associated with persistent wheezing in childhood. *American Journal of Respiratory and Critical Care Medicine*.

[35] Runyon R. S., Cachola L. M., Rajeshuni N. (2012). Asthma discordance in twins is linked to epigenetic modifications of T cells. *PLoS One*.

[36] Yang I. V., Pedersen B. S., Liu A. (2015). DNA methylation and childhood asthma in the inner city. *The Journal of Allergy and Clinical Immunology*.

[37] Curtin J. A., Simpson A., Belgrave D., Semic-Jusufagic A., Custovic A., Martinez F. D. (2013). Methylation of IL-2 promoter at birth alters the risk of asthma exacerbations during childhood. *Clinical and Experimental Allergy*.

[38] Arathimos R., Suderman M., Sharp G. C. (2017). Epigenome-wide association study of asthma and wheeze in childhood and adolescence. *Clinical Epigenetics*.

[39] Sehmi R., Smith S. G., Kjarsgaard M. (2016). Role of local eosinophilopoietic processes in the development of airway eosinophilia in prednisone-dependent severe asthma. *Clinical and Experimental Allergy*.

[40] Barton S. J., Ngo S., Costello P. (2017). DNA methylation of Th2 lineage determination genes at birth is associated with allergic outcomes in childhood. *Clinical and Experimental Allergy*.

[41] Naumova A. K., al Tuwaijri A., Morin A. (2013). Sex- and age-dependent DNA methylation at the 17q12-q21 locus associated with childhood asthma. *Human Genetics*.

[42] Pascual M., Suzuki M., Isidoro-Garcia M. (2011). Epigenetic changes in B lymphocytes associated with house dust mite allergic asthma. *Epigenetics*.

[43] Somineni H. K., Zhang X., Biagini Myers J. M. (2016). Ten-eleven translocation 1 (*TET1*) methylation is associated with childhood asthma and traffic-related air pollution. *Journal of Allergy and Clinical Immunology*.

[44] Tsang M. S., Sun X., Wong C. K. (2020). The role of new IL-1 family members (IL-36 and IL-38) in atopic dermatitis, allergic asthma, and allergic rhinitis. *Current Allergy and Asthma Reports*.

[45] Gagné-Ouellet V., Guay S. P., Boucher-Lafleur A. M., Bouchard L., Laprise C. (2015). DNA methylation signature of interleukin 1 receptor type II in asthma. *Clinical Epigenetics*.

[46] Gao L., Millstein J., Siegmund K. D. (2017). Epigenetic regulation of AXL and risk of childhood asthma symptoms. *Clinical Epigenetics*.

[47] Breton C. V., Byun H. M., Wang X., Salam M. T., Siegmund K., Gilliland F. D. (2011). DNA methylation in the arginase-nitric oxide synthase pathway is associated with exhaled nitric oxide in children with asthma. *American Journal of Respiratory and Critical Care Medicine*.

[48] Baccarelli A., Rusconi F., Bollati V. (2012). Nasal cell DNA methylation, inflammation, lung function and wheezing in children with asthma. *Epigenomics*.

[49] Fu A., Leaderer B. P., Gent J. F., Leaderer D., Zhu Y. (2012). An environmental epigenetic study of ADRB2 5′-UTR methylation and childhood asthma severity. *Clinical and Experimental Allergy*.

[50] Gaffin J. M., Raby B. A., Petty C. R. (2014). *β*-2 adrenergic receptor gene methylation is associated with decreased asthma severity in inner-city schoolchildren: asthma and rhinitis. *Clinical and Experimental Allergy*.

[51] Sood A., Petersen H., Blanchette C. M. (2012). Methylated genes in sputum among older smokers with asthma. *Chest*.

[52] Forno E., Wang T., Qi C. (2019). DNA methylation in nasal epithelium, atopy, and atopic asthma in children: a genome-wide study. *The Lancet Respiratory Medicine*.

[53] Peng C., Cardenas A., Rifas-Shiman S. L. (2018). Epigenome-wide association study of total serum immunoglobulin E in children: a life course approach. *Clinical Epigenetics*.

[54] Stefanowicz D., Hackett T. L., Garmaroudi F. S. (2012). DNA methylation profiles of airway epithelial cells and PBMCs from healthy, atopic and asthmatic children. *PLoS One*.

[55] Gunawardhana L. P., Gibson P. G., Simpson J. L., Benton M. C., Lea R. A., Baines K. J. (2014). Characteristic DNA methylation profiles in peripheral blood monocytes are associated with inflammatory phenotypes of asthma. *Epigenetics*.

[56] Nicodemus-Johnson J., Myers R. A., Sakabe N. J. (2016). DNA methylation in lung cells is associated with asthma endotypes and genetic risk. *JCI Insight*.

[57] Nicodemus-Johnson J., Naughton K. A., Sudi J. (2016). Genome-wide methylation study identifies an IL-13-induced epigenetic signature in asthmatic airways. *American Journal of Respiratory and Critical Care Medicine*.

[58] He W., Kang X., du H. Z. (2014). Defining differentially methylated regions specific for the acquisition of pluripotency and maintenance in human pluripotent stem cells via microarray. *PLoS One*.

[59] Martin E. M., Fry R. C. (2018). Environmental influences on the epigenome: exposure-associated DNA methylation in human populations. *Annual Review of Public Health*.

[60] Joubert B. R., Felix J. F., Yousefi P. (2016). DNA methylation in newborns and maternal smoking in pregnancy: genome-wide consortium meta-analysis. *American Journal of Human Genetics*.

[61] Chhabra D., Sharma S., Kho A. T. (2014). Fetal lung and placental methylation is associated with in utero nicotine exposure. *Epigenetics*.

[62] Suter M. A., Abramovici A. R., Griffin E. (2015). In utero nicotine exposure epigenetically alters fetal chromatin structure and differentially regulates transcription of the glucocorticoid receptor in a rat model. *Birth Defects Research. Part A, Clinical and Molecular Teratology*.

[63] Gruzieva O., Xu C. J., Breton C. V. (2017). Epigenome-wide meta-analysis of methylation in children related to prenatal NO2 air pollution exposure. *Environmental Health Perspectives*.

[64] Rider C. F., Carlsten C. (2019). Air pollution and DNA methylation: effects of exposure in humans. *Clinical Epigenetics*.

[65] Ding R., Jin Y., Liu X. (2016). Characteristics of DNA methylation changes induced by traffic-related air pollution. *Mutation Research, Genetic Toxicology and Environmental Mutagenesis*.

[66] Perera F., Tang W. Y., Herbstman J. (2009). Relation of DNA methylation of 5′-CpG island of ACSL3 to transplacental exposure to airborne polycyclic aromatic hydrocarbons and childhood asthma. *PLoS One*.

[67] Liu J., Ballaney M., al-alem U. (2008). Combined inhaled diesel exhaust particles and allergen exposure alter methylation of T helper genes and IgE production in vivo. *Toxicological Sciences*.

[68] Koh H. Y., Kim T. H., Sheen Y. H. (2019). Serum heavy metal levels are associated with asthma, allergic rhinitis, atopic dermatitis, allergic multimorbidity, and airflow obstruction. *The Journal of Allergy and Clinical Immunology: In Practice*.

[69] Jung K. H., Torrone D., Lovinsky-Desir S. (2017). Short-term exposure to PM2.5 and vanadium and changes in asthma gene DNA methylation and lung function decrements among urban children. *Respiratory Research*.

[70] van der Plaat D. A., de Jong K., de Vries M. (2018). Occupational exposure to pesticides is associated with differential DNA methylation. *Occupational and Environmental Medicine*.

[71] Huang Y. J., Boushey H. A. (2014). The microbiome and asthma.. *Annals of the American Thoracic Society*.

[72] Jackson D. J., Makrinioti H., Rana B. M. (2014). IL-33-dependent type 2 inflammation during rhinovirus-induced asthma exacerbations in vivo. *American Journal of Respiratory and Critical Care Medicine*.

[73] Lund R. J., Osmala M., Malonzo M. (2018). Atopic asthma after rhinovirus-induced wheezing is associated with DNA methylation change in the SMAD3 gene promoter. *Allergy*.

[74] Shang Y., Das S., Rabold R., Sham J. S., Mitzner W., Tang W. Y. (2013). Epigenetic alterations by DNA methylation in house dust mite-induced airway hyperresponsiveness. *American Journal of Respiratory Cell and Molecular Biology*.

[75] Li J. Y., Zhang Y., Lin X. P. (2016). Association between DNA hypomethylation at IL13 gene and allergic rhinitis in house dust mite-sensitized subjects. *Clinical and Experimental Allergy*.

[76] Kim J. S., Shin I. S., Shin N. R., Nam J. Y., Kim C. (2020). Genome-wide analysis of DNA methylation and gene expression changes in an ovalbumin-induced asthma mouse model. *Molecular Medicine Reports*.

[77] Montrose L., Ward T. J., Semmens E. O., Cho Y. H., Brown B., Noonan C. W. (2017). Dietary intake is associated with respiratory health outcomes and DNA methylation in children with asthma. *Allergy, Asthma & Clinical Immunology*.

[78] Mandaviya P. R., Joehanes R., Brody J. (2019). Association of dietary folate and vitamin B-12 intake with genome-wide DNA methylation in blood: a large-scale epigenome-wide association analysis in 5841 individuals. *The American Journal of Clinical Nutrition*.

[79] Shorey-Kendrick L. E., McEvoy C. T., Ferguson B. (2017). Vitamin C prevents offspring DNA methylation changes associated with maternal smoking in pregnancy. *American Journal of Respiratory and Critical Care Medicine*.

[80] Zhang Y., Leung D. Y., Goleva E. (2014). Anti-inflammatory and corticosteroid-enhancing actions of vitamin D in monocytes of patients with steroid-resistant and those with steroid-sensitive asthma. *Journal of Allergy and Clinical Immunology*.

[81] Kidd C. D., Thompson P. J., Barrett L., Baltic S. (2016). Histone modifications and asthma. The interface of the epigenetic and genetic landscapes. *American Journal of Respiratory Cell and Molecular Biology*.

[82] Rothbart S. B., Strahl B. D. (2014). Interpreting the language of histone and DNA modifications. *Biochimica et Biophysica Acta*.

[83] Allfrey V. G., Faulkner R., Mirsky A. E. (1964). Acetylation and methylation of histones and their possible role in the regulation of RNA synthesis. *Proceedings of the National Academy of Sciences of the United States of America*.

[84] Hebbes T. R., Thorne A. W., Crane-Robinson C. (1988). A direct link between core histone acetylation and transcriptionally active chromatin. *The EMBO Journal*.

[85] Jin B., Li Y., Robertson K. D. (2011). DNA methylation: superior or subordinate in the epigenetic hierarchy?. *Genes & Cancer*.

[86] Yang I. V., Schwartz D. A. (2011). Epigenetic control of gene expression in the lung. *American Journal of Respiratory and Critical Care Medicine*.

[87] Barnes P. J. (2009). Histone deacetylase-2 and airway disease. *Therapeutic Advances in Respiratory Disease*.

[88] Cui Z. L., Gu W., Ding T. (2013). Histone modifications of Notch1 promoter affect lung CD4+ T cell differentiation in asthmatic rats. *International Journal of Immunopathology and Pharmacology*.

[89] Li C. Y., Peng J., Ren L. P. (2013). Roles of histone hypoacetylation in LAT expression on T cells and Th2 polarization in allergic asthma. *Journal of Translational Medicine*.

[90] Wang Y., Tian Y., Morley M. P. (2013). Development and regeneration of Sox2+ endoderm progenitors are regulated by a Hdac1/2-Bmp4/Rb1 regulatory pathway. *Developmental Cell*.

[91] Rowell E., Wilson C. B. (2009). Programming perpetual T helper cell plasticity. *Immunity*.

[92] Harb H., Raedler D., Ballenberger N. (2015). Childhood allergic asthma is associated with increased IL-13 and FOXP3 histone acetylation. *The Journal of Allergy and Clinical Immunology*.

[93] Cheng Q., Shang Y., Huang W., Zhang Q., Li X., Zhou Q. (2019). p300 mediates the histone acetylation of *ORMDL3* to affect airway inflammation and remodeling in asthma. *Int Immunopharmacol*.

[94] Stefanowicz D., Lee J. Y., Lee K. (2015). Elevated H3K18 acetylation in airway epithelial cells of asthmatic subjects. *Respiratory Research*.

[95] Clifford R. L., Patel J. K., John A. E. (2015). CXCL8 histone H3 acetylation is dysfunctional in airway smooth muscle in asthma: regulation by BET. *American Journal of Physiology-Lung Cellular and Molecular Physiology*.

[96] Brook P. O., Perry M. M., Adcock I. M., Durham A. L. (2015). Epigenome-modifying tools in asthma. *Epigenomics*.

[97] Sundar I. K., Nevid M. Z., Friedman A. E., Rahman I. (2014). Cigarette smoke induces distinct histone modifications in lung cells: implications for the pathogenesis of COPD and lung cancer. *Journal of Proteome Research*.

[98] Ito K., Lim S., Caramori G., Chung K. F., Barnes P. J., Adcock I. M. (2001). Cigarette smoking reduces histone deacetylase 2 expression, enhances cytokine expression, and inhibits glucocorticoid actions in alveolar macrophages. *The FASEB Journal*.

[99] Nguyen T., Li G. E., Chen H., Cranfield C. G., McGrath K. C., Gorrie C. A. (2018). Maternal E-cigarette exposure results in cognitive and epigenetic alterations in offspring in a mouse model. *Chemical Research in Toxicology*.

[100] Svanes C., Koplin J., Skulstad S. M. (2016). Father's environment before conception and asthma risk in his children: a multi-generation analysis of the Respiratory Health In Northern Europe study. *International Journal of Epidemiology*.

[101] Veremchuk L. V., Tsarouhas K., Vitkina T. I. (2018). Impact evaluation of environmental factors on respiratory function of asthma patients living in urban territory. *Environmental Pollution*.

[102] Vrijens K., Trippas A. J., Lefebvre W. (2020). Association of prenatal exposure to ambient air pollution with circulating histone levels in maternal cord blood. *JAMA Network Open*.

[103] Zheng Y., Sanchez-Guerra M., Zhang Z. (2017). Traffic-derived particulate matter exposure and histone H3 modification: a repeated measures study. *Environmental Research*.

[104] Zakzuk J., Acevedo N., Harb H. (2020). IgE levels to ascaris and house dust mite allergens are associated with increased histone acetylation at key type-2 immune genes. *Front Immunol*.

[105] Brand S., Teich R., Dicke T. (2011). Epigenetic regulation in murine offspring as a novel mechanism for transmaternal asthma protection induced by microbes. *Journal of Allergy and Clinical Immunology*.

[106] Park J., Kim M., Kang S. G. (2015). Short-chain fatty acids induce both effector and regulatory T cells by suppression of histone deacetylases and regulation of the mTOR-S6K pathway. *Mucosal Immunology*.

[107] Thio C. L., Chi P. Y., Lai A. C. Y., Chang Y. J. (2018). Regulation of type 2 innate lymphoid cell-dependent airway hyperreactivity by butyrate. *Journal of Allergy and Clinical Immunology*.

[108] Liu S. K., Ma L. B., Yuan Y. (2020). Alanylglutamine relieved asthma symptoms by regulating gut microbiota and the derived metabolites in mice. *Oxidative Medicine and Cellular Longevity*.

[109] Kolberg L., Forster F., Gerlich J. (2020). Nickel allergy is associated with wheezing and asthma in a cohort of young German adults: results from the SOLAR study. *ERJ Open Research*.

[110] Jose C. C., Wang Z., Tanwar V. S., Zhang X., Zang C., Cuddapah S. (2019). Nickel-induced transcriptional changes persist post exposure through epigenetic reprogramming. *Epigenetics Chromatin*.

[111] Chen H., Kluz T., Zhang R., Costa M. (2010). Hypoxia and nickel inhibit histone demethylase JMJD1A and repress Spry2 expression in human bronchial epithelial BEAS-2B cells. *Carcinogenesis*.

[112] Harb H., Amarasekera M., Ashley S. (2016). Epigenetic regulation in early childhood: a miniaturized and validated method to assess histone acetylation. *International Archives of Allergy and Immunology*.

[113] Alipoor S. D., Adcock I. M., Garssen J. (2016). The roles of miRNAs as potential biomarkers in lung diseases. *European Journal of Pharmacology*.

[114] Ameis D., Khoshgoo N., Iwasiow B. M., Snarr P., Keijzer R. (2017). MicroRNAs in lung development and disease. *Paediatric Respiratory Reviews*.

[115] Weidner J., Ekerljung L., Malmhäll C., Miron N., Rådinger M. (2020). Circulating microRNAs correlate to clinical parameters in individuals with allergic and non-allergic asthma. *Respiratory Research*.

[116] Wardzyńska A., Pawełczyk M., Rywaniak J., Kurowski M., Makowska J. S., Kowalski M. L. (2020). Circulating microRNAs and T-cell cytokine expression are associated with the characteristics of asthma exacerbation. *llergy, Asthma & Immunology Research*.

[117] Wu C., Xu K., Wang Z. (2019). A novel microRNA miR-1165-3p as a potential diagnostic biomarker for allergic asthma. *Biomarkers*.

[118] Lu T. X., Hartner J., Lim E. J. (2011). MicroRNA-21 limits in vivo immune response-mediated activation of the IL-12/IFN-gamma pathway, Th1 polarization, and the severity of delayed-type hypersensitivity. *Journal of Immunology*.

[119] Kim R. Y., Horvat J. C., Pinkerton J. W. (2017). MicroRNA-21 drives severe, steroid-insensitive experimental asthma by amplifying phosphoinositide 3-kinase-mediated suppression of histone deacetylase 2. *The Journal of Allergy and Clinical Immunology*.

[120] Elbehidy R. M., Youssef D. M., el-Shal A. S. (2016). MicroRNA-21 as a novel biomarker in diagnosis and response to therapy in asthmatic children. *Molecular Immunology*.

[121] Lyu B., Wei Z., Jiang L., Ma C., Yang G., Han S. (2020). MicroRNA-146a negatively regulates IL-33 in activated group 2 innate lymphoid cells by inhibiting IRAK1 and TRAF6. *Genes and Immunity*.

[122] Kivihall A., Aab A., Soja J. (2019). Reduced expression of miR-146a in human bronchial epithelial cells alters neutrophil migration. *Clinical and Translational Allergy*.

[123] Polikepahad S., Knight J. M., Naghavi A. O. (2010). Proinflammatory Role for *let-7* MicroRNAS in Experimental Asthma. *The Journal of Biological Chemistry*.

[124] Panganiban R. P., Pinkerton M. H., Maru S. Y., Jefferson S. J., Roff A. N., Ishmael F. T. (2012). Differential microRNA epression in asthma and the role of miR-1248 in regulation of IL-5. *American Journal of Clinical and Experimental Immunology*.

[125] Collison A., Herbert C., Siegle J. S., Mattes J., Foster P. S., Kumar R. K. (2011). Altered expression of microRNA in the airway wall in chronic asthma: miR-126 as a potential therapeutic target. *BMC Pulmonary Medicine*.

[126] Malmhäll C., Alawieh S., Lu Y. (2014). MicroRNA-155 is essential for T_H_2-mediated allergen- induced eosinophilic inflammation in the lung. *Journal of Allergy and Clinical Immunology*.

[127] Arroyo M., Salka K., Chorvinsky E. (2020). Airway miR-155 responses are associated with TH1 cytokine polarization in young children with viral respiratory infections. *PLoS One*.

[128] Korde A., Ahangari F., Haslip M. (2020). An endothelial microRNA-1-regulated network controls eosinophil trafficking in asthma and chronic rhinosinusitis. *The Journal of Allergy and Clinical Immunology*.

[129] Takyar S., Vasavada H., Zhang J. G. (2013). VEGF controls lung Th2 inflammation via the miR-1-Mpl (myeloproliferative leukemia virus oncogene)-P-selectin axis. *The Journal of Experimental Medicine*.

[130] Cho S., Wu C. J., Yasuda T. (2016). miR-23∼27∼24 clusters control effector T cell differentiation and function. *The Journal of Experimental Medicine*.

[131] Yu B., Yao L., Liu C., Tang L., Xing T. (2019). Upregulation of microRNA-16 alters the response to inhaled *β*-agonists in patients with asthma though modulating expression of ADRB2. *Molecular Medicine Reports*.

[132] Lin J., Feng X., Zhang J. (2020). Circular RNA circHIPK3 modulates the proliferation of airway smooth muscle cells by miR-326/STIM1 axis. *Life Sciences*.

[133] Simpson L. J., Patel S., Bhakta N. R. (2014). A microRNA upregulated in asthma airway T cells promotes T_H_2 cytokine production. *Nature Immunology*.

[134] Sun Q., Liu L., Wang H. (2017). Constitutive high expression of protein arginine methyltransferase 1 in asthmatic airway smooth muscle cells is caused by reduced microRNA-19a expression and leads to enhanced remodeling. *Journal of Allergy and Clinical Immunology*.

[135] Nakano T., Inoue Y., Shimojo N. (2013). Lower levels of hsa-mir-15a, which decreases *VEGFA*, in the CD4^+^ T cells of pediatric patients with asthma. *Journal of Allergy and Clinical Immunology*.

[136] Ye L., Mou Y., Wang J., Jin M. L. (2017). Effects of microRNA-19b on airway remodeling, airway inflammation and degree of oxidative stress by targeting TSLP through the Stat3 signaling pathway in a mouse model of asthma. *Oncotarget*.

[137] Lou L., Tian M., Chang J., Li F., Zhang G. (2020). miRNA-192-5p attenuates airway remodeling and autophagy in asthma by targeting MMP-16 and ATG7. *Biomedicine & Pharmacotherapy*.

[138] Dong X., Zhong N., Fang Y., Cai Q., Lu M., Lu Q. (2018). MicroRNA 27b-3p modulates SYK in pediatric asthma induced by dust mites. *Frontiers in Pediatrics*.

[139] Kärner J., Wawrzyniak M., Tankov S. (2017). Increased microRNA-323-3p in IL-22/IL-17-producing T cells and asthma: a role in the regulation of the TGF-*β* pathway and IL-22 production. *Allergy*.

[140] Lu Y., Li Z., Xie B., Song Y., Ye X., Liu P. (2019). hsa-miR-20a-5p attenuates allergic inflammation in HMC-1 cells by targeting HDAC4. *Molecular Immunology*.

[141] Liu J. H., Li C., Zhang C. H., Zhang Z. H. (2020). LncRNA-CASC7 enhances corticosteroid sensitivity via inhibiting the PI3K/AKT signaling pathway by targeting miR-21 in severe asthma. *Pulmonology*.

[142] Tian M., Ji Y., Wang T., Zhang W., Zhou Y., Cui Y. (2018). Changes in circulating microRNA-126 levels are associated with immune imbalance in children with acute asthma. *International Journal of Immunopathology and Pharmacology*.

[143] Mayoral R. J., Deho L., Rusca N. (2011). miR-221 influences effector functions and actin cytoskeleton in mast cells. *PLoS One*.

[144] Shi J., Chen M., Ouyang L. (2020). miR-142-5p and miR-130a-3p regulate pulmonary macrophage polarization and asthma airway remodeling. *Immunology and Cell Biology*.

[145] Johansson K., Malmhäll C., Ramos-Ramírez P., Rådinger M. (2017). MicroRNA-155 is a critical regulator of type 2 innate lymphoid cells and IL-33 signaling in experimental models of allergic airway inflammation. *Journal of Allergy and Clinical Immunology*.

[146] Chung S., Lee Y. G., Karpurapu M. (2020). Depletion of microRNA-451 in response to allergen exposure accentuates asthmatic inflammation by regulating Sirtuin2. *American Journal of Physiology. Lung Cellular and Molecular Physiology*.

[147] Zhao H., Song X., Yan L. (2018). IgE induces hypotension in asthma mice by down-regulating vascular NCX1 expression through activating miR-212-5p. *Biochimica et Biophysica Acta - Molecular Basis of Disease*.

[148] Ong J., van den Berg A., Faiz A. (2019). Current smoking is associated with decreased expression of miR-335-5p in parenchymal lung fibroblasts. *Int J Mol Sci*.

[149] Schembri F., Sridhar S., Perdomo C. (2009). MicroRNAs as modulators of smoking-induced gene expression changes in human airway epithelium. *Proceedings of the National Academy of Sciences of the United States of America*.

[150] Singh S. P., Chand H. S., Langley R. J. (2017). Gestational exposure to sidestream (secondhand) cigarette smoke promotes transgenerational epigenetic transmission of exacerbated allergic asthma and bronchopulmonary dysplasia. *Journal of Immunology*.

[151] Gao L., Liu X., Millstein J. (2018). Self-reported prenatal tobacco smoke exposure, AXL gene-body methylation, and childhood asthma phenotypes. *Clin Epigenetics*.

[152] Liu Q., Wang W., Jing W. (2019). Indoor air pollution aggravates asthma in Chinese children and induces the changes in serum level of miR-155. *International Journal of Environmental Health Research*.

[153] Fry R. C., Rager J. E., Bauer R. (2014). Air toxics and epigenetic effects: ozone altered microRNAs in the sputum of human subjects. *American Journal of Physiology-Lung Cellular and Molecular Physiology*.

[154] Wei Y., Han B., Dai W. (2020). Exposure to ozone impacted Th1/Th2 imbalance of CD4+ T cells and apoptosis of ASMCs underlying asthmatic progression by activating lncRNA PVT1-miR-15a-5p/miR-29c-3p signaling. *Aging (Albany NY)*.

[155] Hirai K., Shirai T., Shimoshikiryo T. (2021). Circulating microRNA-15b-5p as a biomarker for asthma-COPD overlap. *Allergy*.

[156] Wang L., Xu J., Liu H., Li J., Hao H. (2019). PM2.5 inhibits SOD1 expression by up-regulating microRNA-206 and promotes ROS accumulation and disease progression in asthmatic mice. *International Immunopharmacology*.

[157] Li P., Wang J., Guo F., Zheng B., Zhang X. (2020). A novel inhibitory role of microRNA-224 in particulate matter 2.5-induced asthmatic mice by inhibiting TLR2. *Journal of Cellular and Molecular Medicine*.

[158] Alharris E., Alghetaa H., Seth R. (2018). Resveratrol attenuates allergic asthma and associated inflammation in the lungs through regulation of miRNA-34a that targets FoxP3 in mice. *Frontiers in Immunology*.

[159] Xu M., Yu Z., Hu F. (2017). Identification of differential plasma miRNA profiles in Chinese workers with occupational lead exposure. *Bioscience Reports*.

[160] Zhao Z., Wang K., Tan S. (2021). MicroRNA-211-mediated targeting of the INHBA-TGF-*β* axis suppresses prostate tumor formation and growth. *Cancer Gene Therapy*.

[161] Tanwar V. S., Zhang X., Jagannathan L., Jose C. C., Cuddapah S. (2019). Cadmium exposure upregulates SNAIL through miR-30 repression in human lung epithelial cells. *Toxicology and Applied Pharmacology*.

[162] Ding E., Zhao Q., Bai Y. (2016). Plasma microRNAs expression profile in female workers occupationally exposed to mercury. *Journal of Thoracic Disease*.

[163] Rupani H., Martinez-Nunez R. T., Dennison P. (2016). Toll-like receptor 7 is reduced in severe asthma and linked to an altered microRNA profile. *American Journal of Respiratory and Critical Care Medicine*.

[164] Du X., Yang Y., Xiao G. (2020). Respiratory syncytial virus infection-induced mucus secretion by down-regulation of miR-34b/c-5p expression in airway epithelial cells. *Journal of Cellular and Molecular Medicine*.

[165] Moheimani F., Koops J., Williams T. (2018). Influenza A virus infection dysregulates the expression of microRNA-22 and its targets; CD147 and HDAC4, in epithelium of asthmatics. *Respiratory Research*.

